# Supplementary material for: Clinical Characteristics and In Vitro Analysis of MYO6 Variants Causing Late-onset Progressive Hearing Loss
Source: Genes (Basel). 2020 Mar 4;11(3):273. doi: 10.3390/genes11030273 (PMC7140843; doi:10.3390/genes11030273)
Supplement: Supplementary file 1 [file genes-11-00273-s001.zip › Suppl./Supplymentary Table 1.pdf]

Mutagenesis primers	
MYO6 c.374C-A F1	AAGACCACATCATGTCTTTGCAATTGCTGA
MYO6 c.374C-A R1	ACATGATGTGGTCTTGTCCCAAGAGA
MYO6 c.604A-G F1	GCAACAATGATAGCAGTCGATTTGGGAAATTTG
MYO6 c.604A-G R1	TGCTATCATTGTTGCGAACAGTCTTCG
MYO6 c.614G-A F1	TAGCAGTCAATTTGGGAAATTTGTAGAAATACAT
MYO6 c.614G-A R1	CCAAATTGACTGCTATTATTGTTGCGAACAGT
MYO6 c.647A-T F1	TTTTAATGTAAAGAGCTCAGTTGTTGG
MYO6 c.647A-T R1	CTCTTTACATTAAAATGTATTTCTACAAATTT
MYO6 c.1367G-A R1	TCAAAATCAGCAATATCTAGGACTCCAATAAA
MYO6 c.1376G-A F1	TATTGCTGATTTTGAGTACTTTGAGCATAACAGT
MYO6 c.1455T-A F1	TTTTTTAAAGAAAGGATTCTGAAGGAGGA
MYO6 c.1455T-A R1	CCTTTCTTTAAAAAATTGTTGAAGTTTTTCA
MYO6 c.2111G-A F1	GCAGGGTGATTACCCATCACGAGCTTCATTTC
MYO6 c.2111G-A R1	GGGTAATCACCCTGCATCAAGTCCAAAACAG
MYO6 c.2438G-C F1	AAAATATCCAGCTGAAGCCTGCATTAA
MYO6 c.2438G-C R1	TCAGCTGGATATTTTATTTTGTTTTTCAATTT
MYO6 c.3746T-C F1	AAGACAGTCTGAAGAAATCTGGGAACGCTGTG
MYO6 c.3746T-C R1	TCTTCAGACTGTCTTGGCAAGATCTCAGC
Sequencing primers	
MYO6 Seq Primer R2	GCGTTGCTGTCGGTTCATTT
Myo6 Seq Primer R3	CGGCCTGGGCCTCATCACTGATGA
Myo6 Seq Primer R4	CTGGATTTGTTCCTGCGTCATCAT
Myo6 Seq Primer R5	GGTTAACCCAAACTTGTAGTCATTTTC
Myo6 Seq Primer R6	GATTCAAGAGACATATGTAAAGCATCAT
Myo6 Seq Primer R7	GTTCAAAACTGTTATGCTCAAAGTACTC
Myo6 Seq Primer R8	GCACATTCTAATAAAATCACCATGGTC
Myo6 Seq Primer R9	CCATAGGATTCAGTCAGGTATCTTAG
MYO6 Seq Primer F2	GCTTTTCGAGACATGAAGGTGC
MYO6 Seq Primer F3	GTATTTAAACCGAGGCTGCACTAG
MYO6 Seq Primer F4	GTGGAGCAAGCAAACAATGCTC
MYO6 Seq Primer F5	CACAAGGATCATTTTCGACTCACT
MYO6 Seq Primer F6	GATGACAAGCCACCACTTTG
MYO6 Seq Primer F7	GAATGTGGCTTTGCAAGAGGAGAC
MYO6 Seq Primer F8	GCATTCAAGCTGAAGTGGAGGCACAG
MYO6 Seq Primer F9	GAAATACTGAAACAGAGCAACGTGCTC
Halotag pFN21A F1	GCCATTAGCCATATTATTCATTGGTT
Halotag pFN21A F2	GCCCCCTATTGACGTCAATGAC
Halotag pFN21A F3	ATCACTAGAAGCTTTATTGCGGTAG
Halotag pFN21A F4	GACCCCCATTATGTGGAAGTC
Halotag pFN21A F5	GAGTTCATCCGCCCTATCCCGA
Halotag pFN21A F6	GCCAAAAGCCTGCCTAACTGCA
Halotag pFN21A F7	CACAACTAGAATGCAGTGAAAAAAAT
Halotag pFN21A F8	CAAATATGTATCCGCTCATGAGACAATA
Halotag pFN21A F9	GCATCTTACGGATGGCATGA
Halotag pFN21A F10	GCAGCACTGGGGCCAGATGG
Halotag pFN21A F11	GAAGCTCCCTCGTGCGCT
Halotag pFN21A F12	GCTCTTGATCCGGCAAACAAACC
Halotag pFN21A F13	CAGTTTTTCGGGTGGTTTGTTGCC
Halotag pFN21A R1	GATCTCGATCCTCTACGCCGG
Halotag pFN21A R2	CGTGAGTTTTCGTTCCACTGAGC
Halotag pFN21A R3	CAGCTTGGAGCGAACGACCTAC
Halotag pFN21A R4	CTTAATCAGTGAGGCACCTATCTC
Halotag pFN21A R5	GTTGTGCAAAAAAGCGGTTAGC
Halotag pFN21A R6	GCAAAATGCCGCAAAAAAGGGA
Halotag pFN21A R7	GTTGTTGTTAACTTGTTTATTGCAGC
Halotag pFN21A R8	GCGATCTCGCTGCCGATCAGGT
Halotag pFN21A R9	GATCAGCTTGCGGCCGACGTCG
Halotag pFN21A R10	GTCGGGTTACCGTGCAGGAAC
Halotag pFN21A R11	GCACTGACTGCGTTAGCAATTTA
Halotag pFN21A R12	GCATATGATACACTTGATGTACTGCC
