# Supplementary figures and images for: Clinical Characteristics and In Vitro Analysis of MYO6 Variants Causing Late-onset Progressive Hearing Loss
Source: Genes (Basel). 2020 Mar 4;11(3):273. doi: 10.3390/genes11030273 (PMC7140843; doi:10.3390/genes11030273)

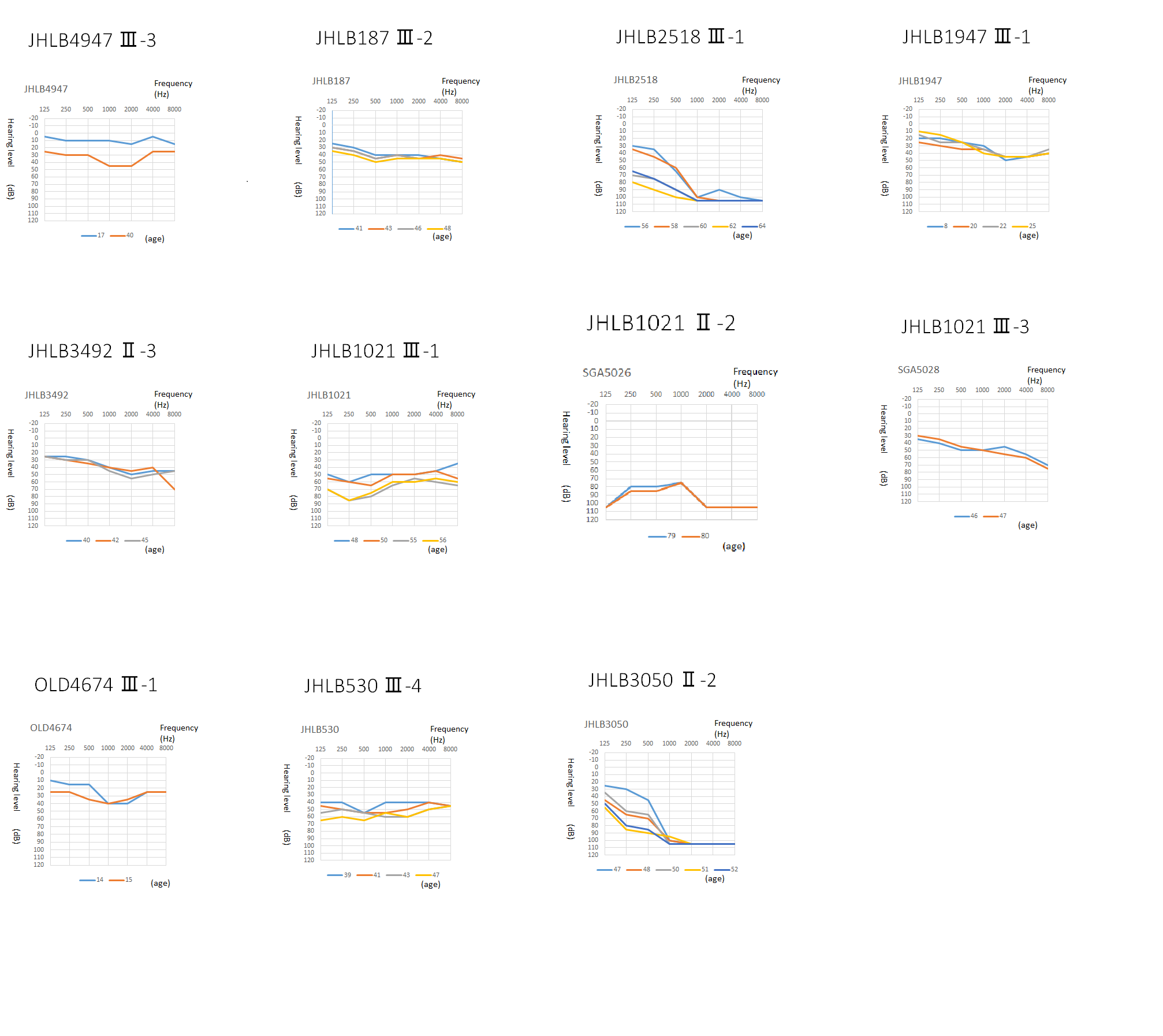

Supplement: Supplementary file 1 [file genes-11-00273-s001.zip › Suppl./Supply figure1 20202-24.png]

A.

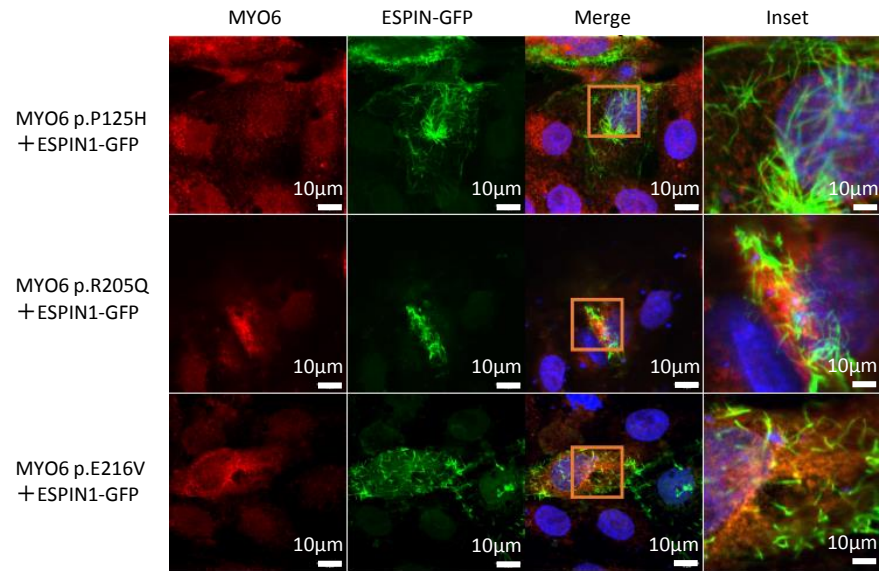

B.

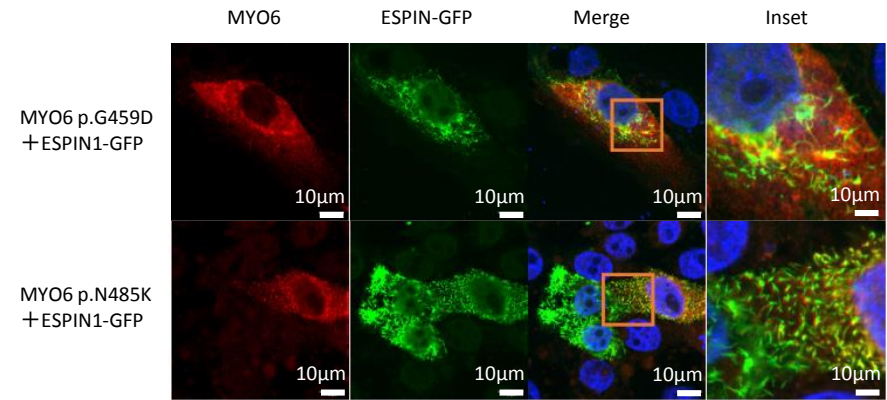

C.

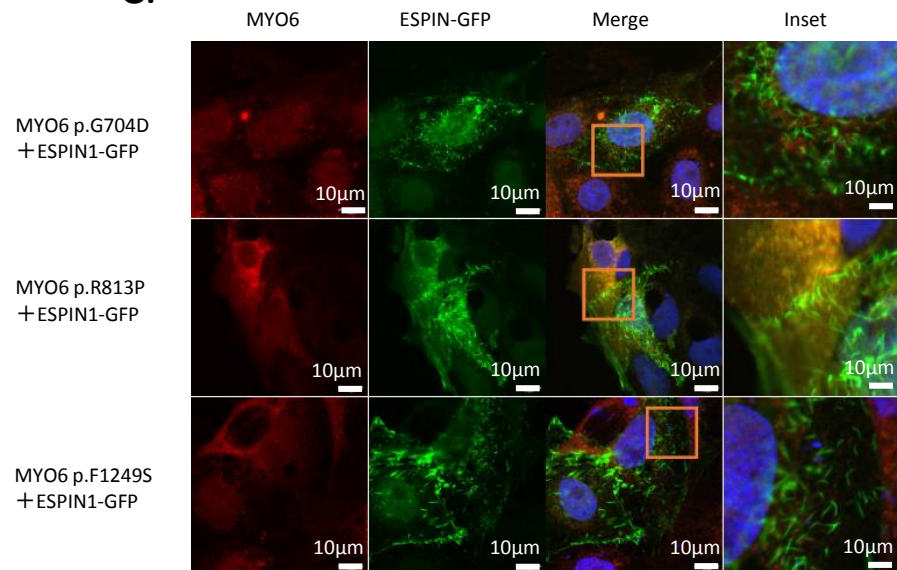

Supplement: Supplementary file 1 [file genes-11-00273-s001.zip › Suppl./Supplymentary Figure 2.pdf]
